# Regulation of *HTT* mRNA Biogenesis: The Norm and Pathology

**DOI:** 10.3390/ijms252111493

**Published:** 2024-10-26

**Authors:** Alexandra E. Zubkova, Dmitry V. Yudkin

**Affiliations:** 1 Federal State Autonomous Educational Institution of Higher Education I.M. Sechenov First Moscow State Medical University of the Ministry of Health of the Russian Federation (Sechenov University), Trubetskaya Str., 8/2, Moscow 119048, Russia; zubkova_a_e@staff.sechenov.ru; 2 Department of Natural Sciences, Novosibirsk State University, Pirogova 2, Novosibirsk 630090, Russia

**Keywords:** Huntington’s disease, CAG repeat, transcriptional regulatory elements, mRNA processing, miRNA, alternative splicing, incomplete splicing

## Abstract

Huntington’s disease (HD) is a neurodegenerative disorder caused by the expansion of the CAG repeat in exon 1 of the *HTT* gene, leading to the formation of a toxic variant of the huntingtin protein. It is a rare but severe hereditary disease for which no effective treatment method has been found yet. The primary therapeutic targets include the mutant protein and the mutant mRNA of *HTT*. Current clinical trial approaches in gene therapy involve the application of splice modulation, siRNA, or antisense oligonucleotides for RNA-targeted knockdown of *HTT*. However, these approaches do not take into account the diversity of *HTT* transcript isoforms in the normal conditions and in HD. In this review, we discuss the features of transcriptional regulation and processing that lead to the formation of various *HTT* mRNA variants, each of which may uniquely contribute to the progression of the disease. Furthermore, understanding the role of known transcription factors of *HTT* in pathology may aid in the development of potentially new therapeutic tools based on endogenous regulators.

## 1. Introduction

Huntington’s disease (HD) is a hereditary neurodegenerative disorder that is widespread worldwide, with a frequency of up to 15 per 100,000 persons [1,2]. The main symptoms include motor disorders and chorea. Additionally, neuropsychiatric deficits, cognitive defects, and memory impairments develop in patients with HD [3]. The basis of these symptoms lies in multiple intracellular changes that lead to cell death. The pathology is most pronounced in neurons of the central nervous system, particularly among the medium spiny neurons of the striatum [3]. On average, the symptoms of the disease manifest between the ages of 30 and 50, but they can appear earlier in cases of juvenile-onset HD [4,5]. Currently, the condition of patients with HD is managed through a wide range of targeted therapy methods [6]. However, an effective etiological treatment method has yet to be found.

The main genetic cause of the development of HD is the expansion of the CAG repeat in exon 1 of the *HTT* gene, which encodes the huntingtin protein. This leads to the formation of a polyglutamine (polyQ) tract at the N-terminus of this protein [3]. The human *HTT* gene is approximately 180 kb size and is located at 4p16.3 locus [7]. This gene is expressed ubiquitously, with a high level of expression characteristic of various types of cells in the central nervous system [8,9]. *HTT* expression is maintained throughout life, but it plays a crucial role during early embryonic development [8]. This is evidenced by embryonic lethality in mice with the huntingtin gene knockout [10].

HD develops at a CAG repeat length of 36 or more triplets. With complete penetrance, the pathological phenotype manifests at 40 or more triplets [8]. A CAG repeat length of 27 or more triplets is genetically unstable, meaning it is prone to either contraction or expansion during transmission between generations [11]. The increase in the number of CAG repeat triplets in the offspring relative to the parental number occurs predominantly via paternal transmission [8,12,13]. In this case, the offspring experience an earlier age of onset of the disease and more rapid disease progression [4,12]. Genetic instability is also observed in somatic tissues, being most pronounced in post-mitotic neurons of the striatum and cortex [14,15].

In HD, many intracellular processes are disrupted, including transcriptional regulation, mitochondrial homeostasis, proteasomal degradation, autophagy, etc. [8,16,17,18]. All of this ultimately leads to cell death [8]. Both the mutant protein and the mutant mRNA of *HTT* contribute to the pathogenesis of the disease. Pathogenic variants of full-length huntingtin with expanded polyQ tracts are prone to forming insoluble cytoplasmic aggregates. These aggregates can bind to other proteins, disrupting their natural function [19]. The main contribution to the development of HD comes from the production of N-terminal huntingtin protein fragments. Their aggregates are primarily localized in the nucleus, where they affect gene expression and the integrity of the nuclear envelope [20,21]. However, recent studies have shown that *HTT* mRNA with an expanded CAG repeat is also prone to interacting with proteins and disrupting their function. For example, they can form cytoplasmic gel-like RNA foci that bind the elongation factor eEF2, thereby disrupting translation [22]. Additionally, these mRNAs can sequester splicing factors [23]. The role of *HTT* mRNA in the pathogenesis of HD is further supported by the fact that the length of the uninterrupted CAG repeat, rather than the length of polyQ, correlates with the age of onset of the disease [24]. However, the full contribution of mutant *HTT* mRNA to the development of toxicity in HD still requires further investigation.

The direct and indirect involvement of *HTT* mRNA with an expanded CAG repeat in the pathogenesis of HD identifies it as a significant target for therapy of this disease. Understanding which factors affect RNA biogenesis in pathology is essential for developing new therapeutic approaches. In this review, we present an analysis of the features of transcriptional regulation and processing of known isoforms of normal and mutant human mRNA of *HTT*.

## 2. Regulation of *HTT* Gene Transcription

### 2.1. Functional Features of the HTT Promoter

The human *HTT* promoter is a CG-enriched sequence lacking TATA and CCAAT motifs [25]. The known functionally significant elements of the promoter are located up to −2100 bp relative to the *HTT* translation start site [25]. The sequence from −206 bp to −56 bp relative to the translation start site is highly conserved, containing two main transcription start sites (TSS) at positions −145 bp and −135 bp. Upstream of the TSS, there are two clusters of tandem direct repeats. Between −516 bp and −483 bp, there are two 17 bp repeats, and between −212 bp and −173 bp, there are two 20 bp repeats [25,26]. The sequence containing the 20 bp repeats likely performs an important regulatory function, as deletion of even one repeat significantly reduces *HTT* expression levels [25,27]. Between −2100 bp and −1812 bp, there is one full *Alu* element, and a 3′ fragment of an *Alu* element is located between −1724 bp and −1598 bp, both oriented opposite to the direction of *HTT* transcription [26]. The effects of these repeats on the regulation of *HTT* expression have not been studied yet.

### 2.2. Transcriptional Regulators of HTT: In the Norm and in HD

**Figure 1:**
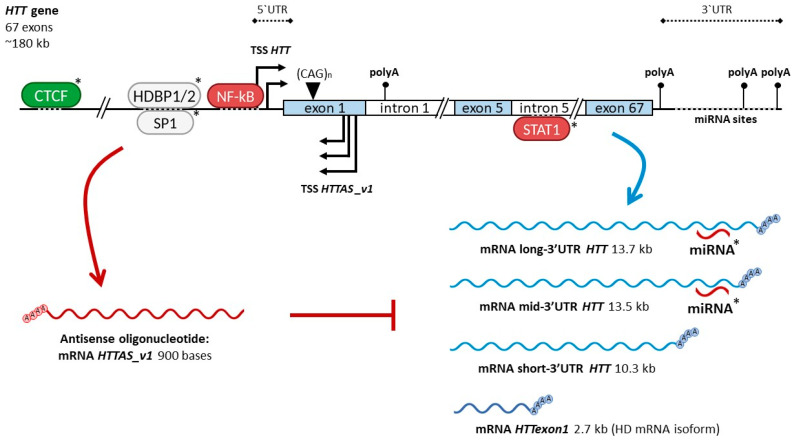
Regulation of *HTT* transcription. The relative location of the regulatory elements at the *HTT* locus is presented. Polyadenylation sites are marked as “polyA”. *HTT* transcriptional regulators with a known influence on gene expression in the norm are indicated: positive regulators as CTCF (green), negative regulators as NF-kB, STAT1, miRNA, and mRNA HTTAS_v1 (red), SP1 and HDBP1/2 having an ambiguous role (gray). The “*” symbol marks regulators with functions that are either unknown in HD or may differ from their roles in the norm. The red arrow denotes the expression of an antisense transcript, the blue arrows denote the expression of sense *HTT* mRNA isoforms. The most common *HTT* mRNA isoforms are shown: full-length long-/mid-/short-3′UTR transcripts and short toxic *HTTexon1* transcript by incomplete splicing. Refer to the text for additional explanations.

Many regulatory elements of the human *HTT* promoter with a determined influence on gene expression are located within the sequence from −1300 bp to the translation start site. Among them are binding sites for regulators such as CTCF, SP1, HDBP1, HDBP2, and NF-kB (Figure 1) [25]. The binding site of the functionally significant transcription factor STAT1 is located in intron 5 of the gene (Figure 1) [25]. The binding sites of known microRNAs regulating *HTT* expression are located in the 3′ untranslated region (3′ UTR) of the gene (Figure 1) [28,29]. Additionally, the level of *HTT* transcription is regulated by the expression of an antisense transcript (Figure 1) [30].

#### 2.2.1. CTCF

- Norm

A CTCF binding site is located in the sequence from −1285 bp to −1270 bp relative to the *HTT* TSS (Figure 1) [25]. This site is tissue-specifically differentially methylated, with lower DNA methylation levels being preferable for CTCF binding. In the cerebral cortex, huntingtin is expressed at a relatively high level, where the methylation level of the CTCF binding site in the *HTT* promoter is relatively low, and the CTCF level is elevated. This underlines CTCF’s role as a positive regulator of *HTT* gene expression (Table 1) [31].

- HD

No changes in DNA methylation patterns associated with HD have been identified, and the involvement of CTCF in the development of the pathology has not been reported (Table 1) [31].

#### 2.2.2. SP1

- Norm

The sequence from −813 bp to −9 bp relative to the *HTT* TSS contains 12 dispersed potential binding sites for the transcription factor SP1 [25]. Normally, this factor acts as a positive regulator of *HTT* transcription (Figure 1) [27,35]. The SP1 binding sites, each located within the 20 bp tandem direct repeats from −212 bp to −173 bp, contribute the most to regulation. Introducing single-nucleotide mutations into the SP1 binding site sequences of these repeats led to a decrease in *HTT* expression levels. A high level of gene expression is maintained only if two tandem repeats are present [27]. However, results from an experiment with HEK293 cells involving siRNA-mediated SP1 knockdown show a modest increase in *HTT* expression [34]. This indicates the ambiguous role of SP1 in regulating *HTT* expression (Table 1).

- HD

In HD, mutant huntingtin can bind SP1, prevent the factor from interacting with promoters, and reduce the expression of SP1-regulated genes [8,38]. Meanwhile, the expression level of SP1 itself is increased. Suppression of SP1 in experiments on transgenic HD mice showed a neuroprotective effect. The mechanism of this effect is still unclear, although it has been shown that SP1 knockdown had a minor impact on reducing mutant huntingtin expression in mouse models (Table 1) [35,36]. No other studies on the role of SP1 in regulating *HTT* expression in HD have been published by the end of 2024.

#### 2.2.3. HDBP1 and HDBP2

- Norm

Transcription factors HDBP1 and HDBP2 are proteins highly homologous to each other and capable of binding to the same sequence in the *HTT* promoter [32]. This sequence is present in three copies: two copies are located within the 20 bp tandem direct repeats from −212 bp to −173 bp relative to the *HTT* TSS, and another copy is located proximal to the 20 bp tandem direct repeats from −220 bp to −214 bp (Figure 1) [25,32].

To test the regulatory activity of these transcription factors, a reporter construct with the luciferase gene sequence driven by a synthetic promoter was used. The synthetic promoter was fused with a fragment of the *HTT* promoter from −231 bp to −146 bp, containing the binding sites for HDBP1 and HDBP2. Mutations introduced in the binding site sequences of these transcription factors led to a decrease in luciferase activity in transfected neuronal IMR32 cell lines (human neuroblastoma) compared to the norm. Meanwhile, a similar experiment in non-neuronal HeLa cells did not show changes in luciferase activity upon mutation introduction compared to the norm. This indicates the role of HDBP1 and HDBP2 as positive regulators of *HTT* transcription in neuronal cells [32]. However, another study suggests that HDBP1 and HDBP2 are repressors of *HTT* expression. In transgenic neuronal-derived U87 MG cells (a human glioblastoma cell line), exogenous expression of HDBP2 led to a decrease in luciferase activity driven by the *HTT* promoter. The exogenous expression of HDBP1 affected luciferase activity to a lesser extent. Thus, the role of HDBP1 and HDBP2 in regulating the *HTT* promoter is ambiguous (Table 1) [33].

- HD

Currently, the influence of HDBP1 and HDBP2 on the regulation of *HTT* expression in HD has not been studied, and no such research has been published to date (Table 1).

#### 2.2.4. NF-kB

- Norm

The transcription factor NF-kB binds to the sequence from −148 bp to −139 bp relative to the *HTT* TSS, which overlaps the TSS at −145 bp (Figure 1) [25]. In HEK293 cells, siRNA-mediated knockdown of NF-kB leads to an increase in *HTT* expression, identifying this factor as a negative regulator of *HTT* gene transcription (Table 1) [34].

- HD

The regulatory single-nucleotide polymorphism rs13102260 (G > A) (rSNP) in the NF-kB binding site leads to the substitution of the −148 nucleotide in the *HTT* promoter, located three nucleotides away from the *HTT* TSS. The presence of this rSNP prevents the binding of NF-kB to the *HTT* promoter and reduces gene expression. In HD patients, the rSNP on the mutant allele is associated with a later age of onset of the disease, while the rSNP on the normal allele is associated with an earlier age of onset. Thus, the presence of the rSNP in the NF-kB binding site influences the relative proportions of the normal and mutant huntingtin in cells with HD (Table 1) [37].

#### 2.2.5. STAT1

- Norm

The binding site for the transcription regulator STAT1 is located in intron 5 of the *HTT* gene (Figure 1) [25]. In HEK293 cells, STAT1 is identified as a negative regulator of *HTT* gene transcription, as siRNA-mediated knockdown of STAT1 increases gene expression (Table 1) [34].

- HD

Currently, the effect of STAT1 on the regulation of *HTT* expression in HD has not been studied (Table 1).

#### 2.2.6. MicroRNAs

- Norm

MicroRNAs (miRNAs) regulate the expression of many human genes [39]. It has been shown that miRNAs can negatively regulate the expression of *HTT*. It is known that miRNA-137, miRNA-148a, miRNA-214, miRNA-150, miRNA-146a, and miRNA-125b target the 3′UTR of *HTT* mRNA (Figure 1) [28,29]. The analysis of the effects of these miRNAs on gene expression was conducted using a reporter construct containing the luciferase gene fused with the *HTT* 3′UTR sequence, which includes the miRNA binding sites. In a model using non-neuronal HEK293T cells containing the reporter construct, exogenous expression of each of miRNA-137, miRNA-148a, and miRNA-214 led to a decrease in luciferase activity. Additionally, exogenous miRNA-137, miRNA-148a, and miRNA-214 suppressed the expression of endogenous *HTT* in HEK293T cells [29]. In another experiment, knock-in mice STHdh^Q7^/Hdh^Q7^ cells were used as the cell model. These are striatal cells expressing the normal variant of huntingtin [40]. Exogenous expression of each of miRNA-214, miRNA-150, miRNA-146a, or miRNA-125b also led to a decrease in luciferase activity in STHdh^Q7^/Hdh^Q7^ cells containing the reporter construct. Additionally, it was shown that the same exogenous miRNA-214, miRNA-150, miRNA-146a, or miRNA-125b suppress the endogenous expression of *HTT* in STHdh^Q7^/Hdh^Q7^ cells [28]. According to these data, at least miRNA-214 is capable of regulating *HTT* expression in both mouse neuronal and human non-neuronal cells (Table 1).

- HD

The expression of many miRNAs, including those regulating *HTT* expression, changes during HD. It is suggested that their differential expression could be used as a disease marker [41,42].

The expression of miRNA-150 and miRNA-125b is reduced in HD knock-in mice STHdh^Q111^/Hdh^Q111^ cells compared to the normal STHdh^Q7^/Hdh^Q7^ cells. Additionally, in cells with an expanded CAG repeat, the expression of miRNA-214 and miRNA-148a is elevated [28]. In the striatum of HD patients, the expression level of miRNA-137 is suppressed compared to the normal level [41]. The literature presents contradictory data on the changes in miRNA-146a expression. The expression of miRNA-146a is reduced in HD STHdh^Q111^/Hdh^Q111^ cells but increased in the HD human striatum [28,41]. Thus, the role of miRNA-146a in disease development has not been established yet. The ambiguous data may be related to the involvement of miRNAs in large-scale gene networks, and consequently, the wide variety of gene targets affected by miRNAs [28]. Therefore, the differential expression of miRNAs in HD is tissue- and species-specific.

Definitive conclusions about the role of individual miRNAs in regulating *HTT* expression in HD have also not been made yet. An analysis of luciferase activity was conducted in STHdh^Q7^/Hdh^Q7^ cells and HD STHdh^Q111^/Hdh^Q111^ cells using a reporter construct containing the luciferase gene sequence fused with the miRNA-146a binding site in the 3′UTR of *HTT*. In transfected HD STHdh^Q111^/Hdh^Q111^ cells, increased luciferase activity was observed compared to the results in STHdh^Q7^/Hdh^Q7^ cells. These results can be explained by the fact that the level of miRNA-146 is reduced in mutant cells relative to the normal level, and thus, the negative regulatory effect of miRNA on gene expression is decreased in mutant cells relative to the normal level [28]. However, further research is needed to interpret the impact of these results on the development of HD, particularly in human HD (Table 1).

#### 2.2.7. Antisense Transcript

- Norm

The antisense transcript of *HTT* (*HTTAS*) is expressed from the *HTT* sequence, has several alternative TSS, and undergoes alternative splicing. Three TSS of *HTTAS* are located distal to the CAG repeat relative to the sense strand within the boundaries of *HTT* exon 1 (Figure 1). Transcription from these TSS and processing of corresponding transcripts result in the *HTTAS_v1* mRNA isoform, about 900 bases in length (Figure 1). It contains two exons: *HTTAS* exon 1 and *HTTAS* exon 3. *HTTAS* exon 1 encodes a CUG repeat and is partially complementary to *HTT* exon 1 and the *HTT* 5′UTR. *HTTAS* exon 3 is complementary to a sequence located much more proximal to the *HTT* promoter on the sense strand. The *HTTAS_v1* isoform predominates in cells. The fourth TSS of *HTTAS* is located in a sequence proximal to the TSS of *HTT* relative to the sense strand and encodes a short mRNA isoform, *HTTAS_v2*, about 800 bases in length. This isoform contains *HTTAS* exon 2, complementary to the *HTT* promoter sequence, and *HTTAS* exon 3 [30].

*HTTAS_v1* is expressed in the cerebral cortex, hippocampus, caudate, and putamen both in the norm and in HD. Induced overexpression of *HTTAS_v1* in non-neuronal HEK293 cells and neuronal SH-SY5Y cells reduced the level of endogenous *HTT*, while siRNA-mediated knockdown of *HTTAS_v1* led to an increase in *HTT* expression [30]. Furthermore, it has been shown that the involvement of *HTTAS_v1* in *HTT* silencing is mediated by the RNA-induced silencing complex (RISC) pathway, which includes Dicer. Both overexpression and low expression of *HTTAS_v1* increased *HTT* expression levels in mouse embryonic stem cells in the absence of Dicer [30]. This confirms the role of *HTTAS_v1* as a negative regulator of *HTT* transcription controlled by the RISC pathway (Figure 1) (Table 1). However, it is currently unknown how the short isoform *HTTAS_v2* affects *HTT* expression [43].

- HD

The expression level of *HTTAS_v1* is reduced in HD brains. It has been suggested, in this case, that the expression of *HTT* would be increased in HD brains. However, *HTT* levels differ minimally between the normal and HD brains. To determine the reasons for the reduced level of *HTTAS_v1* in HD, reporter constructs driven by the *HTT* promoter were used. These constructs had a single open reading frame (ORF) containing the exon 1 sequence with varying sizes of the CAG repeat, the endogenous *HTTAS_v1* promoter, and the *GFP* gene sequence. Studies in HEK293 cells containing the reporter construct showed that an expanded CAG repeat correlates with reduced expression of *HTTAS_v1* but does not affect *HTT* expression levels. However, when a construct with the endogenous *HTTAS_v1* promoter replaced by a strong CMV promoter was used, the expression level of *HTTAS_v1* increased, and *HTT* was correspondingly reduced, compared to the experiment with the complete absence of the *HTTAS_v1* promoter. This occurred regardless of the CAG repeat length [30]. These results are supported by experiments in mouse Dicer null embryonic stem cells. With low *HTTAS_v1* expression in the absence of Dicer, *HTT* expression was increased only with a normal CAG repeat length. This indirectly suggests that low *HTTAS_v1* expression does not influence *HTT* expression regulation in HD. In the case of overexpression of *HTTAS_v1* in the absence of Dicer, *HTT* expression was increased regardless of the CAG repeat length, whereas in the presence of Dicer, *HTT* expression was reduced in this scenario [30]. Thus, exogenous upregulation of *HTTAS_v1* expression may help reduce levels of mutant *HTT* in HD (Table 1).

## 3. *HTT* mRNA Processing

### 3.1. Diversity of HTT Transcripts Due to Alternative Polyadenylation Signals

- Norm

The full-length *HTT* transcript contains 67 exons including an *HTT* coding sequence (CDS) of approximately 9.5 kb [7]. Currently, three full-length RNA isoforms of *HTT* are known. These isoforms are formed by alternative polyadenylation in the 3′UTR (Figure 1) [44]. The isoform of about 13.7 kb in length (long-3′UTR *HTT*) is predominantly present in non-dividing cells, particularly in the brain, breast, and ovary [44,45]. The isoform of about 13.5 kb in length (mid-3′UTR *HTT*) is highly expressed in the brain and ovary [45]. The shortest isoform (short-3′UTR *HTT*) of about 10.3 kb in length predominates in dividing cells in the testes, B-cells, and muscle [44,45].

- HD

Changes in the relative abundance of alternative polyadenylation *HTT* isoforms are characteristic of the HD motor cortex and cerebellum. A relative decrease in long-3′UTR *HTT* and an increase in mid-3′UTR *HTT* and short-3′UTR *HTT* are observed in the HD motor cortex. A relative decrease in short-3′UTR occurs in the HD cerebellum. Meanwhile, the total expression level of full-length *HTT* is reduced compared to the physiological normal levels in the HD motor cortex and remains unchanged in the HD cerebellum [44].

The relative increase in short-3′UTR *HTT* in the HD motor cortex is associated with disease progression [44]. Longer isoforms with a greater 3′UTR length are more likely to contain binding sites for miRNA and other potential negative regulators. Thus, the expression level of long isoforms may be reduced in HD [46].

The reduction in the expression of full-length *HTT* specifically in the motor cortex is also associated with disease progression. In HD, there is an increase in the relative level of the short *HTT* transcript, which is synthesized as a result of incomplete splicing of *HTT* (more details about incomplete splicing are presented in Section 3.2, under “HD”) [47].

### 3.2. Diversity of HTT Transcripts Due to Alternative Splicing

- Norm

The wide diversity of human *HTT* transcript variants is formed not only through the variety of alternative polyadenylation isoforms but also through alternative splicing. In addition to the canonical full-length isoform containing 67 exons, the mRNA *HTT-Δ29* isoform, lacking exon 29, is characteristic of the adult healthy human brain (Table 2) [48]. Additionally, the mRNA *HTT-Δ28* isoform, lacking exon 28, is formed in the human brain. However, the deletion of exon 28 results in a frameshift, and such a transcript may undergo nonsense-mediated mRNA decay during translation. The presence or absence of the protein encoded by the mRNA *HTT-Δ28* has not been studied [48]. Analysis of *HTT-Δ28* and *HTT-Δ29* transcripts using PCR with specific primers showed product presence only after reamplification, indicating low levels of these transcripts in adult human brain cells. No correlation between the presence of these isoforms and the development of HD was found [48]. Additionally, an mRNA *HTT-Δ34–44* isoform, lacking the region from exon 34 to exon 44, was previously described in the human brain [49]. However, further studies on this transcript have not been conducted. In human embryonic stem cells (hESCs), other alternatively spliced mRNA variants were also identified: *HTT-Δ10*, lacking exon 10; *HTT-Δ12*, *HTT-Δ13*, and *HTT-Δ46*, partially lacking exons 12, 13, and 46, respectively; and *HTT-41b*, characterized by an additional exon 41b (Table 2). All of these isoforms are found at low levels both in the normal conditions and in HD [50]. The mRNA isoforms *HTT-Δ28* and *HTT-Δ34–44* were not detected in hESCs [50]. These mRNA variants, except for *HTT-Δ28*, are spliced in the ORF, meaning they can be completely translated. However, the absence or presence of additional domains in the final protein variant may affect the function of huntingtin, adding or removing sites for post-translational modifications, and altering susceptibility to proteolysis and caspase-dependent cleavage [48,50]. The functional changes in the mRNA characteristic of some isoforms will be presented in more detail herein.

The mRNA isoform *HTT-Δ29*, identified in the adult human brain, lacks exon 29 (Table 2). A bioinformatic analysis of the sequence and structure of the region encoded by exon 29 revealed homology with part of the TAP protein binding site sequence. TAP mediates the nuclear transport of mRNA [52]. Deletion of exon 29 leads to a disruption in interaction with TAP [48]. However, the impact of exon 29 deletion on the function of huntingtin has not been experimentally established, nor has the role of the TAP binding site in huntingtin’s function.

The mRNA isoform *HTT-Δ10*, identified in hESCs, lacks exon 10, which encodes amino acids 427 to 442 (Table 2). It is known that amino acid Ser434 is phosphorylated in the full-length isoform, but it is absent in the variant encoded by *HTT-Δ10* mRNA [50]. This phosphorylation is associated with reduced caspase-dependent cleavage of huntingtin. Typically, short N-terminal forms of huntingtin, resulting from proteolysis, are associated with the progression of HD [53]. Thus, the *HTT-Δ10* mRNA isoform may encode a relatively more toxic variant than full-length huntingtin (Table 2) [50]. Interestingly, unlike other known *HTT* mRNA isoforms, *HTT-Δ10* is expressed irregularly throughout development, with its expression decreasing during neural differentiation [50]. The *HTT-Δ10* mRNA isoforms in the adult brain have not been studied.

The mRNA isoform *HTT-Δ12*, identified in hESCs, lacks 135 nucleotides from the 3′ end of exon 12, which encode amino acids 539 to 583 (Table 2). This splicing variant results in the loss of the caspase cleavage site at D552 [50,54]. Consequently, the protein variant in this case will be more resistant to proteolysis and less toxic to the cell than its full-length alternative. Thus, the *HTT-Δ12* mRNA isoform should be more resistant to the development of HD (Table 2) [50]. However, experiments confirming this hypothesis have not been conducted yet.

The mRNA isoform *HTT-Δ13*, identified in hESCs, lacks 48 nucleotides from the 3′ end of exon 13 (Table 2). The mRNA isoform *HTT-Δ46* lacks 30 nucleotides from the 5′ end of exon 46 (Table 2). The amino acid sequences encoded by the missing regions do not contain any known sites of post-translational modifications. The effect of *HTT-Δ13* and *HTT-Δ46* mRNA on the structure and function of huntingtin has not been studied yet (Table 2) [50].

The mRNA isoform *HTT-41b* includes an additional exon 41b inserted between exons 41 and 42 (Table 2). Sequence analysis revealed that this new exon is formed through the insertion of an *Alu* element [50]. The *HTT-41b* mRNA isoform has been found not only in hESCs but also in the human adult brain cortex (Table 2) [55]. However, the potential sites of post-translational modification in the amino acid sequence encoded by exon 41b, as well as its impact on the structure and function of huntingtin, have not been studied yet.

Thus, all currently known isoforms formed as a result of alternative splicing of *HTT* mRNA are characteristic of both the normal conditions and HD [48,50]. The *HTT-Δ10* mRNA isoform might be associated with HD; however, it appears to be expressed only during the early stages of embryonic development, before the onset of disease progression (Table 2) [50]. Further research is needed to understand the potential role of different mRNA isoforms on the structure and function of huntingtin.

- HD

There is an mRNA *HTT* isoform predominantly specific to HD. This is the short transcript *HTTexon1*, consisting of the sequences 5′UTR, exon 1 of *HTT*, and part of intron 1 of *HTT* (Figure 1). It was previously thought that it might form due to incomplete splicing caused by the activation of cryptic polyadenylation sites at positions 2710 bp or 7327 bp within intron 1 of *HTT* (the length of intron 1 is about 12 kb) [56]. However, in subsequent studies on fibroblasts from HD patients with an expanded CAG repeat, only the 7327 bp polyadenylation site was found [51]. The 2710 bp polyadenylation site was found to be specific to the YAC128 mouse model, which contains the full-length human *HTT* gene with an expanded CAG repeat [51,57].

The production of *HTTexon1* mRNA has been detected in mouse models containing the full-length *HTT* gene, in human fibroblasts from HD patients, and in postmortem brain cortex samples from adults, particularly in the cerebellum, sensory motor cortex, and hippocampus (Table 2) [51,56]. In all cases, the CAG repeat length was more than 67 triplets, corresponding to juvenile-onset HD [51,56]. Besides the juvenile form of the disease, genetic instability of the CAG repeat in somatic tissues may lead to the formation of *HTTexon1* mRNA [51]. The incomplete splicing resulting in the formation of *HTTexon1* mRNA is a consequence of the relatively low elongation rate of PolII in *HTT* gene variants with an expanded CAG repeat [58]. Moreover, a longer CAG repeat is associated with higher expression levels of *HTTexon1* mRNA [51,58]. This correlation can be explained by a cotranscriptional “window of opportunity” model between transcription and processing processes. In other words, slow elongation rates promote the activation of a less competitive but earlier localized alternative processing site, which would be outcompeted by a more competitive site during faster elongation [59]. In the case of *HTTexon1* transcription, the low elongation rate may be related to the aberrant sequestration of splicing factors on the expanded CAG repeat [60]. With slow elongation, PolII is more sensitive to the cryptic polyadenylation site in intron 1, leading to premature termination of transcription [21,60]. Thus, the involvement of the SRSF6 factor in modulating incomplete splicing was demonstrated using a model system of minigene expression in HEK293 cells, which represent sequences of fragments from the mouse huntingtin gene. Changes in SRSF6 expression levels affected the possibility of incomplete splicing in the presence of a CAG repeat length of 100 triplets [58]. Other experiments in mouse models showed that reducing SRSF6 levels does not affect incomplete splicing, which may indicate the involvement of other splicing factors in the in vivo processing of *HTTexon1* mRNA [21,61].

The mRNA isoform *HTTexon1* is translated into a truncated N-terminal fragment of huntingtin, encoded only by exon 1 of *HTT*. The unspliced exon 1 is followed by a stop codon, resulting in the formation of the huntingtin exon 1 protein, which ends with a proline residue in all vertebrates [51,56]. The huntingtin exon 1 protein is associated with severe HD progression, specifically participating in the formation of nuclear and cytoplasmic aggregates [21,51]. The aggregates of the N-terminal mutant huntingtin formed in the nucleus can recruit various transcription factors, leading to dysregulation of gene expression in HD [62]. These N-terminal mutant variants potentially include huntingtin exon 1 proteins [21].

## 4. Discussion

HD develops as a result of the expression of a mutant allele of the *HTT* gene with an expanded CAG repeat [3]. It is known that both the mutant huntingtin protein and the mutant *HTT* mRNA influence the development of the disease [19,22]. Additionally, factors regulating the transcription of *HTT* and the diversity of produced *HTT* mRNA isoforms contribute to the pathology [25,44,47,48,50]. All these can serve as potential targets for the development of HD therapy. Therefore, understanding the features of normal and pathological transcription of the *HTT* gene is of direct significance for the development of both targeted and etiological methods of HD therapy.

The positive regulators of *HTT* transcription include CTCF (Figure 1, Table 1) [25,31]. However, this role in disease development has not been defined. The involvement of the HDBP1, HDBP2, and SP1 factors in the regulation of *HTT* expression is ambiguous (Table 1) [32,33,34,35]. Nevertheless, SP1’s role as a positive regulator is suggested by the fact that during disease progression, a reduction in SP1 levels improves HD patients’ condition by slightly lowering huntingtin levels [36]. However, the regulation via SP1 under HD may also be influenced by other factors. The role of HDBP1 and HDBP2 in HD is still unknown. However, HDBP1, HDBP2, and SP1 share binding site locations within the same sequence, located within the 20 bp tandem direct repeats from −212 bp to −173 relative to the *HTT* TSS. Since mutations in the binding sites for HDBP1, HDBP2, and SP1 have led to reduced *HTT* expression in experiments, searching for HD-specific SNPs in these sequences might help determine the role of these factors in the disease. Negative regulators of *HTT* transcription include NF-kB and STAT1, but experimental data indicating participation in HD pathogenesis have only been obtained for NF-kB (Figure 1, Table 1) [34,37]. In the *HTT* promoter, an rSNP in the NF-kB binding site has been described, which reduces the affinity of this factor for DNA. This leads to decreased *HTT* expression and, if the rSNP is on the mutant allele, alleviation of the disease course (Table 1) [37]. However, considering the normal function of NF-kB as a repressor, the reduction in *HTT* expression due to impaired factor function raises questions. It is possible that the role of rSNP is more about disrupting the interaction of other components of the transcriptional machinery with the *HTT* promoter, since the rSNP binding site overlaps with the TSS of *HTT*. Thus, it is challenging to find potential candidates among protein regulators for the role of a therapeutic agent in HD.

Non-coding RNAs, such as miRNA-137, miRNA-148a, miRNA-214, miRNA-150, miRNA-146a, miRNA-125b, and the long non-coding RNA *HTTAS*, also act as repressors of *HTT* transcription [28,29,30]. The involvement of miRNAs in large genetic networks disrupted in HD complicates determining the role of these molecules in regulating *HTT* expression in pathology (Table 1). In the case of *HTTAS*, induced overexpression of the *HTTAS_v1* isoform antisense transcript in HD reduced the expression of the sense *HTT*, mediated by the endogenous RISC pathway (Figure 1) [30]. This identifies *HTTAS_v1* as a potential therapeutic regulator in the development of the pathology (Table 1).

Full-length *HTT* mRNA has three known isoforms due to alternative polyadenylation, with a higher possibility of shorter isoforms forming in HD [44]. In addition to alternative polyadenylation, the diversity of *HTT* mRNA isoforms is generated through alternative splicing [47,48,50]. At least eight alternatively spliced, translatable isoforms are known (Table 2). The presence of four of these, namely, the main *HTT* mRNA isoform consisting of 67 exons, *HTT-Δ29*, *HTT-41b*, and the short isoform *HTTexon1* formed as a result of incomplete splicing, has been experimentally confirmed in the adult human brain [48,51,55]. *HTTexon1* is the most pathogenic isoform, but it is present in cells only in cases of large expansion of the CAG repeat longer than 67 triplets (Table 2) [51,56]. This mRNA produces N-terminal huntingtin protein fragments, which have increased neurotoxicity [56]. Currently, various antibody options targeting this protein are being developed, but an antibody-based approach has not been proposed for clinical trials yet [21]. The presence of *HTT-Δ10* and *HTT-Δ12* isoforms has been confirmed only in hESCs, but these variants could potentially modulate the progression of HD in the case of an expanded CAG repeat [50]. *HTT-Δ10* contains a deletion of a phosphorylation site associated with a decreased chance of huntingtin proteolysis, leading to the formation of toxic short N-terminal forms. Accordingly, the production of *HTT-Δ10* from the mutant allele may result in a more severe course of the disease (Table 2). Conversely, *HTT-Δ12* contains a deletion of a proteolysis site, meaning that the production of this isoform from the mutant allele may reduce the chance of forming a toxic protein variant (Table 2) [50].

Currently, splicing modulation is one of the most promising methods of HD therapy [63,64]. Among all the potentially possible *HTT* mRNA isoforms, many variants exist in very low abundance in the cells due to the mechanism of nonsense-mediated mRNA decay. Normally, the stop codon is located at the end of ORF in the last exon and no stop codons can be located in other coding parts of the mRNA. However, if alternative splicing results in an isoform with a stop codon in an intermediate exon, such mRNA will undergo degradation [65]. The drug PTC518, which is included in clinical trials (NCT05358717), induces the inclusion of a pseudoexon containing a premature stop codon, leading to the degradation of the corresponding mRNA [64]. Another relevant therapeutic approach is the development of synthetic siRNAs and antisense oligonucleotides. These molecules are capable of participating in processes silencing *HTT* expression, such as mRNA degradation or blocking protein translation [64]. Currently included in clinical trials are the siRNA-based drug AMT-130 (NCT04120493, NCT05243017) and antisense oligonucleotide therapies Tominersen and VO659 (NCT05686551, NCT05822908) [64]. Among the aforementioned drugs, PTC518, AMT-130, and Tominersen are allele-nonspecific, whereas VO659 preferentially targets the expanded CAG repeat of mutant *HTT* mRNA. VO659 and AMT-130 also target *HTTexon1* mRNA, whereas Tominersen and PTC518 do not target this shortened transcript [64]. The effectiveness of these drugs in reducing huntingtin expression is determined by measuring the level of mutant protein in the human cerebrospinal fluid (CSF). The most successful in this regard so far is Tominersen; however, the final efficacy results for the other drugs have not yet been obtained [64]. It should be noted that trials for VO659 (NCT05822908) and AMT-130 (NCT05243017) are still in the recruitment phase, while Tominersen is the most studied drug. The Generation-HD1 trial for Tominersen (NCT03761849) was completed in 2022 but did not pass safety checks. In the CSF analysis of patients, inflammation markers were found, which are attributed more to the pro-inflammatory effects of antisense oligonucleotides than to the reduction of huntingtin levels [64]. Currently, the Generation-HD2 trial, aimed at determining an effective and safe dose of Tominersen (NCT05686551), is in the recruitment phase. Thus, the safety of these drugs currently in clinical trials remains an important issue. Despite the potential benefits of developing the above approaches in gene therapy of HD, all of them are potentially effective only in the case of the adult-onset form before or during the early manifestation of HD symptoms [64]. In this review, we describe potentially the most toxic *HTT* mRNA isoforms, such as the short-3′UTR polyadenylation isoform and *HTTexon1*, as well as *HTT-Δ10* in the case of the mutant allele. Optimizing the application of non-coding RNAs or splice modulation regarding the most toxic *HTT* mRNA isoforms may have therapeutic potential in HD therapy.

## Figures and Tables

**Table 1:** Regulation of *HTT* gene expression by transcriptional regulators.

Transcriptional Regulators	Expected Effect on *HTT* Expression	
	In the Norm	In HD
CTCF	Positive [31]	Unknown
HDBP1/2	Ambiguous [32,33]	Unknown
SP1	Ambiguous [27,34]	Negative [35,36]
NF-kB	Negative [34]	Negative * [37]
STAT1	Negative [34]	Unknown
miRNA	Negative [28,29]	Ambiguous [28,29]
*HTTAS*	Negative [30]	Negative [30]

* In the case of rSNP (see explanations in the text).

**Table 2:** Diversity of *HTT* mRNA alternative splicing isoforms.

*HTT* mRNA Isoforms	Expected HD Effect
Full-length *HTT*	Ambiguous [8]
*HTTexon1*	Toxic [51]
*HTT-Δ29*	Unknown [48]
*HTT-41b*	Unknown [50]
*HTT-Δ10*	Toxic * [50]
*HTT-Δ12*	Neuroprotective * [50]
*HTT-Δ13*	Unknown [50]
*HTT-Δ46*	Unknown [50]

* Without confirming experiments (see explanations in the text).
